# *PLA2G6* ‐associated late‐onset parkinsonism in a Sudanese family

**DOI:** 10.1002/acn3.51781

**Published:** 2023-05-03

**Authors:** Yousuf Bakhit, Christelle Tesson, Mohamed O. Ibrahim, Khalid Eltom, Isra Eltazi, Liena E.O. Elsayed, Suzanne Lesage, Osheik Seidi, Jean‐Christophe Corvol, Ullrich Wüllner, Sudanese Parkinson's Disease Study Group

**Affiliations:** ^1^ Department of Neurology University Hospital Bonn Bonn Germany; ^2^ Department of Basic Medical Sciences, Faculty of Dentistry University of Khartoum Khartoum Sudan; ^3^ Sudan Neuroscience Projects University of Khartoum Khartoum Sudan; ^4^ Assistance Publique Hôpitaux de Paris, Department of Neurology, Pitié‐Salpêtrière Hospital Sorbonne Université, Paris Brain Institute – ICM, Inserm, CNRS Paris France; ^5^ Department of Biochemistry, Faculty of Medicine Sudan University of Science and Technology Khartoum Sudan; ^6^ Department of Medical Cell Biology, Uppsala Biomedical Center Uppsala University Uppsala Sweden; ^7^ Department of Neurology, Soba Teaching Hospital, And Department of Medicine, Faculty of Medicine University of Khartoum Khartoum Sudan; ^8^ Department of Basic Sciences, College of Medicine Princess Nourah bint Abdulrahman University Riyadh Saudi Arabia; ^9^ German Center for Neurodegenerative Diseases (DZNE) Bonn Germany

## Abstract

**Introduction:**

The phospholipase A2 group VI gene (*PLA2G6*) encodes an enzyme that catalyzes the hydrolytic release of fatty acids from phospholipids. Four neurological disorders with infantile, juvenile, or early adult‐onset are associated with *PLA2G6* genetic alterations, namely infantile neuroaxonal dystrophy (INAD), atypical neuroaxonal dystrophy (ANAD), dystonia‐parkinsonism (DP), and autosomal recessive early‐onset parkinsonism (AREP). Few studies in Africa reported *PLA2G6*‐associated disorders and none with parkinsonism of late adult onset.

**Material and Methods:**

The patients were clinically assessed following UK Brain Bank diagnostic criteria and International Parkinson and Movement Disorder Society's Unified Parkinson's Disease Rating Scale (MDS‐UPDRS). Brain MRI without contrast was performed. Genetic testing was done using a custom‐made Twist panel, screening 34 known genes, 27 risk factors, and 8 candidate genes associated with parkinsonism. Filtered variants were PCR‐amplified and validated using Sanger sequencing and also tested in additional family members to study their segregation.

**Result:**

Two siblings born to consanguineous parents developed parkinsonism at the age of 58 and 60 years, respectively. MRI showed an enlarged right hippocampus in patient 2, but no overt abnormalities indicative of INAD or iron deposits. We found two heterozygous variants in *PLA2G6*, an in‐frame deletion NM_003560:c.2070_2072del (p.Val691del) and a missense variant NM_003560:c.956C>T (p.Thr319Met). Both variants were classified as pathogenic.

**Conclusion:**

This is the first case in which *PLA2G6* is associated with late‐onset parkinsonism. Functional analysis is needed to confirm the dual effect of both variants on the structure and function of iPLA2β.

## Introduction

The Phospholipase A2 group VI gene (*PLA2G6*) encodes an enzyme that catalyzes the hydrolytic release of fatty acids from phospholipids, producing lysophospholipids, which are used in deacetylation/reacetylation reactions. It possesses a calcium‐dependent phospholipase activity, which has a role in phospholipid remodeling, an attribute consistent with cellular and mitochondrial membrane integrity and signal transduction.^1^ It is expressed in the brain and the gene product, iPLA2‐β, is abundant in nerve terminals and neuronal dendrites.^2^ It has been demonstrated that decreased levels of iPLA2‐β disrupt the skeletal structure of the Golgi complex,^3^ trafficking from the Golgi complex to the endoplasmic reticulum,^4^, ^5^ and impair Vps26 and Vps35 levels, retromer functions and ceramides levels in Parkinson's Disease (PD) models, similar to α‐synuclein associated neurodegeneration.^2^

According to the age of onset and clinical features, *PLA2G6*‐associated neurodegeneration (PLAN) can be categorized into 4 phenotypes: infantile neuroaxonal dystrophy (INAD), atypical neuroaxonal dystrophy (ANAD), adult‐onset dystonia‐parkinsonism (DP), and autosomal recessive early‐onset parkinsonism (AREP). Symptoms of INAD and ANAD appear in infancy to early childhood and manifest mainly as progressive psychomotor deterioration, ataxia, spasticity, and axial dystonia.^6^, ^7^ Cerebellar atrophy and iron deposition in the basal ganglia are common radiological signs which are detected in most cases using magnetic resonance imaging (MRI). Therefore, PLAN could be classified as Neurodegeneration with Brain Iron Accumulation (NBIA).^8^, ^9^

DP usually occurs between 20 and 40 years of age and manifests with parkinsonism, dystonia, and sometimes epilepsy. Earlier presentations in the second decade of life are also documented. In addition, impaired cognitive functions are common phenomena, and iron deposition incidence is scant.^10^, ^11^ The disease has a rapid progression and responds well to levodopa and dopamine receptor agonists, and early‐onset dyskinesia is common in patients who receive levodopa.^12^

In the early stages of their disease, *PLA2G6*‐associated AREP patients exhibit bradykinesia, lower limb tremor, hypomimia, and gait disturbance. With the progression of the symptoms, additional features are observed in some cases, such as dystonia, dysarthria, dysphagia, cognitive impairment, and extrapyramidal signs and symptoms. Non‐parkinsonian features may develop at later stages and may encompass cerebellar ataxia and autonomic dysfunction. Similar to DP, iron accumulation in the brain is rare. Instead, frontal and generalized white matter atrophy may be observed.^13^, ^14^ Moreover, a recent comprehensive article revealed that in some cases, mild to moderated cerebral and cerebellar atrophy was observed with a degree of variability in several newly identified cases.^15^ Limb tremor and gait disturbance have favorable responses to dopaminergic agents.

To the best of our knowledge, no cases of adult‐onset *PLA2G6*‐associated parkinsonism have been reported in Africa. Here, we introduce the first case of genetically confirmed autosomal recessive parkinsonism with a late‐onset disease in the sixth and seventh decades of life, identified in two siblings from a Sudanese family.

## Material and Methods

### Patients

A clinical diagnosis of PD was made in 2 patients using the United Kingdom Parkinson's Disease Society Brain Bank criteria^16^ and confirmed using the International Parkinson and Movement Disorder Society's Unified Parkinson's Disease Rating Scale (MDS‐UPDRS).^17^ T1‐ and T2‐weighted MRI brain scans were obtained for both patients without intravenous contrast. No T2* or SWI scans were performed.

The Research Ethics committee for Medical and Health studies at the Faculty of Medicine, University of Khartoum, Sudan, approved the study. Written informed consent was obtained from all participants.

### Sampling

Five individuals (two patients, a healthy mother, and two healthy siblings) donated saliva for DNA extraction. One to two milliliters of saliva were collected using Oragene^®^ Discover. DNA purification was carried out following prepIT^®^ L2P manual protocol. DNA quality and quantity were performed using agarose gel electrophoresis and spectrophotometry (Nanodrop^®^, Thermoscientific).

### Genetic testing

We utilized Twist custom panel, which allows screening of 34 genes, 27 risk variants, and 8 candidate genes, which are mostly associated with Parkinsonism (panel details are available in the Table S1). The custom Twist EF Library Prep (Twist) was used to capture all exons, intron– exon boundaries, 5′‐ and 3′‐UTR sequences and 10‐bp flanking sequences of target genes (RefSeq database, hg38 assembly). Specific probes for NGS target enrichment were designed using Twist software, and amplicon length varied between 250 and 500 bp. Runs were performed on Illumina MiSeq sequencer. The assay was performed according to the manufacturer's recommended protocol. Variants were prioritized based on the following criteria: frequencies <0.01% in public databases (ExAC/GnomAD) and our in‐house database of 500 exomes, nucleotide and amino‐acid conservation (based on alignments), relation of the gene to disease (per family), and inheritance pattern conservation (based on alignments).

### Bioinformatics analysis of gene panel data

Human reference genome UCSC hg19 was used for sequence alignment and variant calling with the Burrows‐Wheeler Aligner (BWA)^18^ and the Genome Analysis Toolkit (GATK).^19^ PCR duplicates were removed prior to variant calling using Picard software. The mean coverage was 993× (range 594–1241×), and the mean percent coverage at 30× was 98.7% (range 96.5–99.6%) for all individuals tested. Variants were annotated with ANNOVAR software.^20^ Variants were prioritized based on the following criteria: frequencies <0.01% in public databases (ExAC/gnomAD) and our in‐house database of 500 exomes, nucleotide and amino‐acid conservation (based on alignments), relation of the gene to disease (per family), and inheritance pattern. We visualized the molecular effect of the missense variant on the structure of iPLA2‐β protein using UCSFC ChimeraX.^21^

### Sanger sequencing

Filtered variants were PCR‐amplified and validated (Sanger sequencing) to confirm the genotypes in affected individuals. The variants were Sanger‐sequenced in three unaffected relatives (the mother, a non‐identical twin to patient 1 and, a brother) to study the segregation. Forward and reverse primers were designed using Primer3 online tool.^22^ Multiple sequence alignment was performed using Bioedit software v7.2.5^23^ using default parameters.

## Results

### Clinical presentation

**Figure 1:**
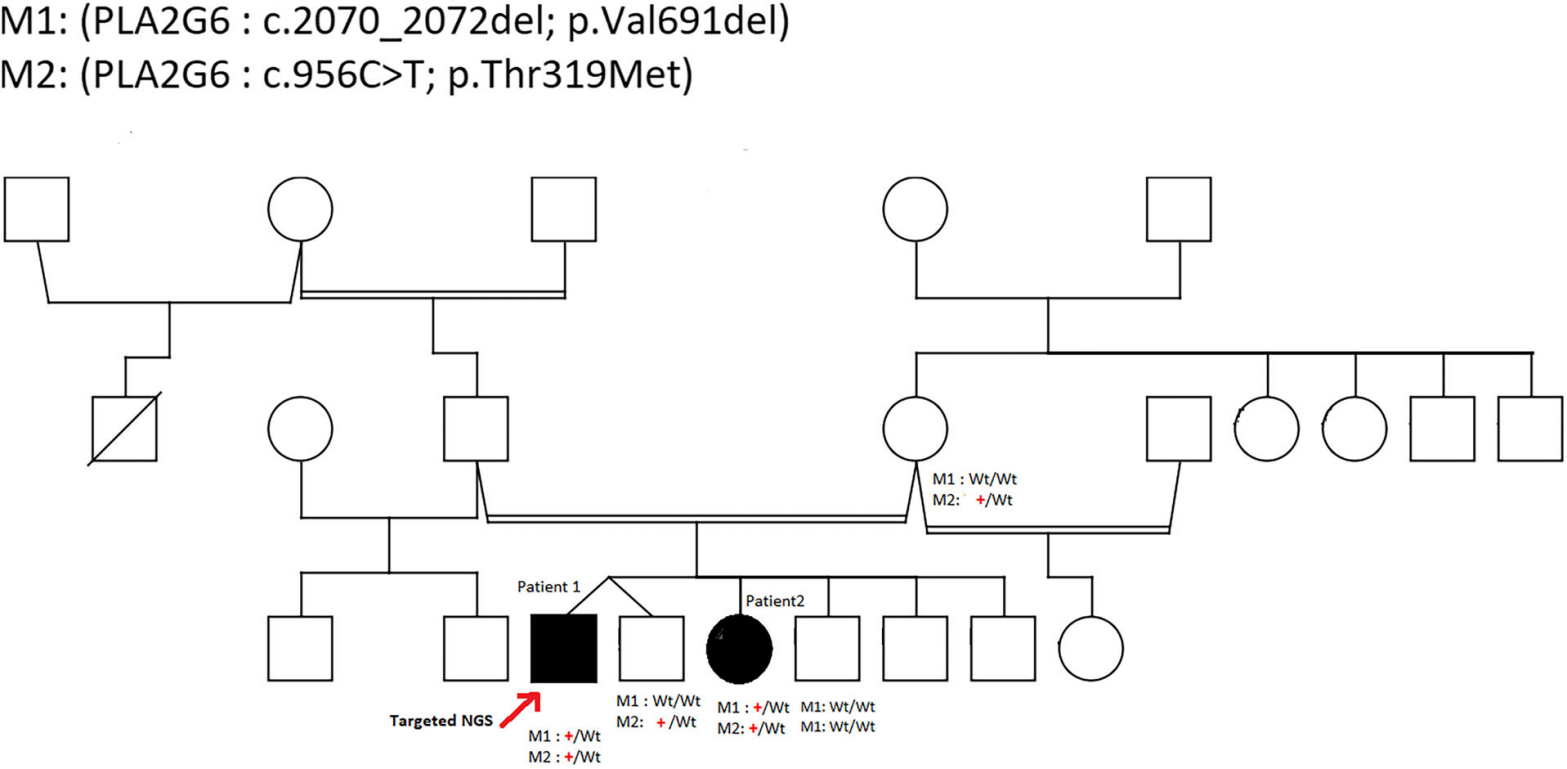
Heredogram showing the segregation result of two siblings diagnosed with autosomal recessive late onset Parkinsonism, their mother and two healthy sibling . the first variant (p.Val691del) was considered M1 while the second variant (p.Thr319Met) is considered M2. The red plus indicates a carrier of the variant while the wild type is indicated with (wt). The red arrow indicated the targeted individual for the panel.

Two siblings born to consanguineous parents (first‐degree cousins; Fig. 1) were diagnosed with PD. In the first patient (patient 1), symptoms appeared at the age of 58 years and started as a resting tremor in his right arm. Within three years, his condition developed into bilateral resting tremor in the upper limbs, with generalized slowness of movement and excessive salivation but with no drooling. Upon assessment, rigidity was detected with activation maneuvers in the neck and all limbs equally. Posture was not quite erect, and a mild disturbance of gait was detected. Symptoms improved with intake of levodopa with only minimal motor fluctuations. No dyskinesia was observed.

The second patient (patient 2) developed the first symptom at the age of 60 years in the form of resting tremor at her left arm. Her condition worsened within five years as the tremor markedly interfered with most of the daily activities, in addition to developing intermittent freezing of gait. Symptoms initially improved with levodopa; however, at the time of examination, the patient suffered from marked end‐of‐dose motor fluctuations and Levodopa‐induced dyskinesia. This patient also reported frequent hallucinations, sleeping problems (mainly insomnia), and inability to control urination. Upon assessment, she had hypomimia (i.e., facial masking), dysarthria and showed symptoms of dopamine dysregulation syndrome.

Upon assessment “on” levodopa, mild bradykinesia was observed in patient 1 while more progressive bradykinesia was observed in patient 2; neither of the two patients showed resting tremor while moderate rigidity in both patients was found in all extremities and neck. We were unable to perform a formal detailed cognitive assessment.

### Investigations and radiographic finding

**Figure 2:**
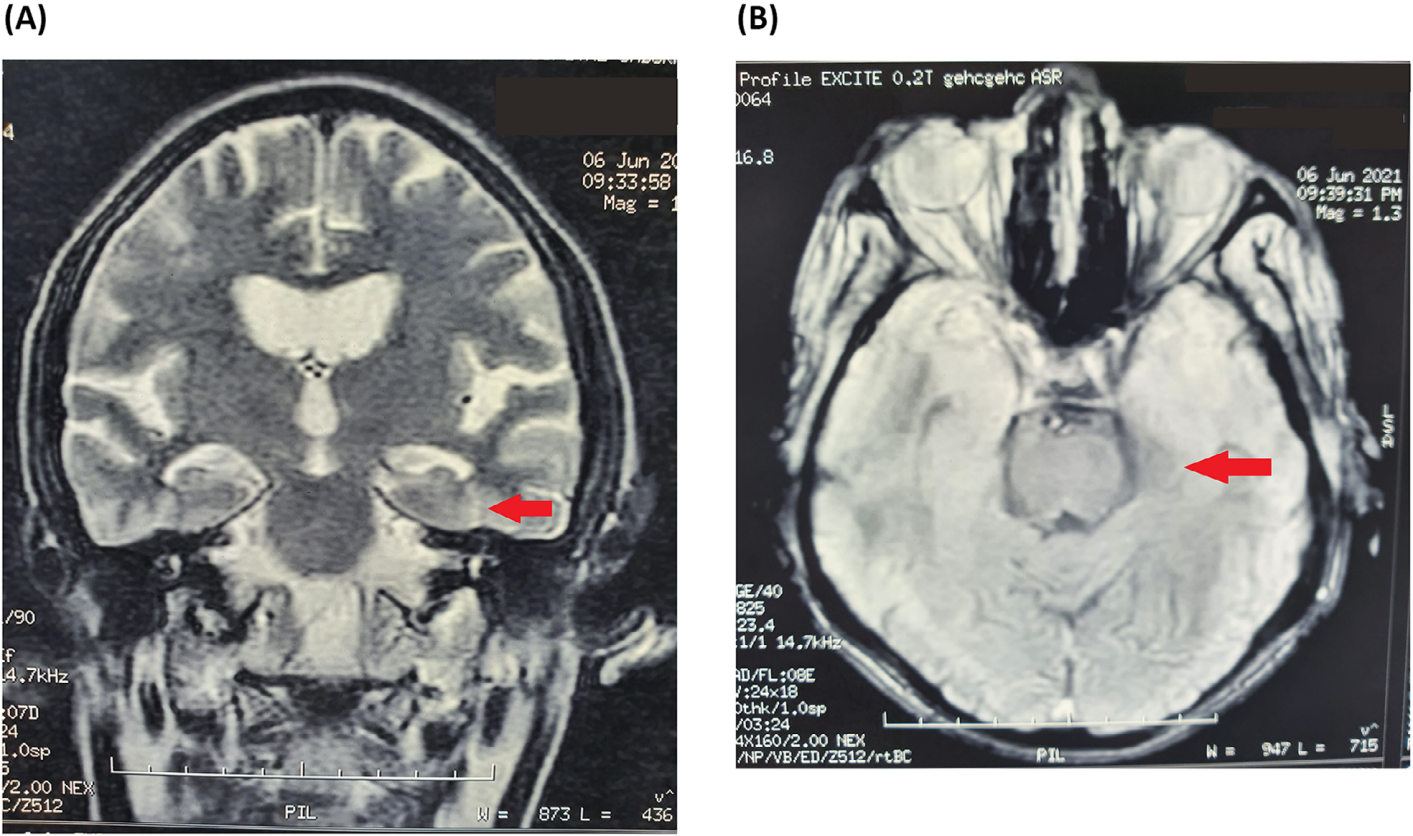
T2‐weighted MRI brain scans of patient 1 showing moderate hippocampal expansion on the left side, as indicated with the red arrow. (A) Coronal section. (B) Horizontal section.

We performed fundoscopy, lipid profile, high‐resolution lipoprotein electrophoresis, peripheral blood smear, serum iron, serum ferritin, and transferrin saturation studies for both patients. Prompted by a history of hearing impairment in patient 2, we performed Ear, Nose, and Throat examination as well as audiometric testing. All these investigations were unremarkable for both patients. We found moderate hippocampal expansion on T2‐weighted MRI brain scans of patient 1 (Fig. 2). Brain MRI was done without intravenous contrast and did not included T2* nor SWI sequences.

### Identified variants using the genetic panel

Two heterozygous variants in *PLA2G6* (NM_003560.4:c.2070_2072del (p.Val691del) and NM_003560.4: c.956C>T (p.Thr319Met)) were found in both siblings (Patient 1 and Patient 2) showing late onset parkinsonism. List of variants found and their pathogenecity scores are found in Table S2. We were also able to analyze copy number variations from the panel which was unremarkable for both patients.

### Analysis of *PLA2G6* variants

**Figure 3:**
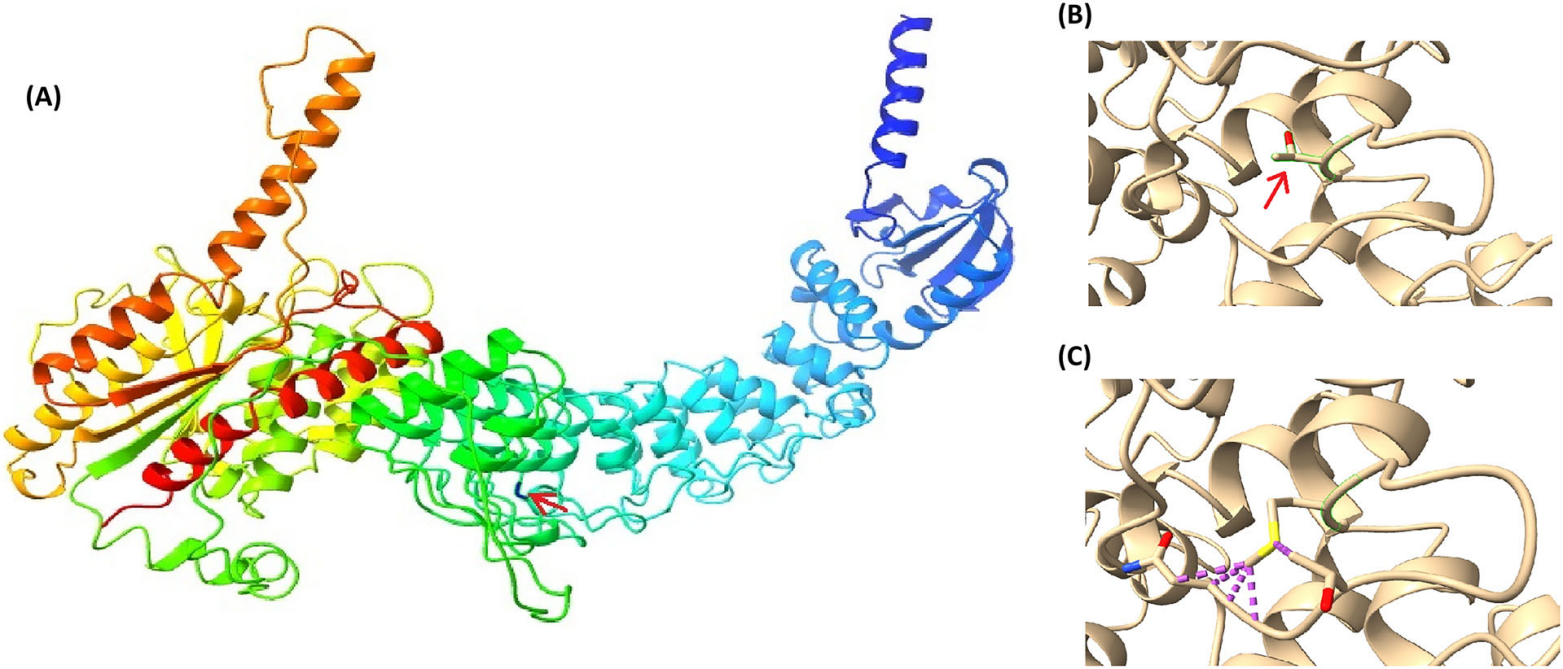
Homology modeling of iPLA2‐β showing the effect of the mutant variant (p.Thr319Met)on the structure of the protein, inflicting at least four clash point (indicated with dotted purple lines) with the surrounding amino acid residues. (A) Three‐dimensional structure of the protein iPLA2‐β, with the site of amino acid changed indicated in red arrow. (B) Wild type residue Threonine. (C) Mutant residue Methionine.

Both variants found in *PLA2G6* were validated using Sanger sequencing. Segregation analysis (Fig. 1) showed that the mother and one healthy sibling were carriers for one variant (p.Thr391Met). A sample from the father—who was deceased at the time of sampling—was not available. Thus, confirming the paternal origin of the other variant (p.Val691del) was not feasible. The mother and two healthy siblings had reference alleles at the genomic location tested (wildtype). Chromatograms of all family members tested are available in Fig. S1. Although a *trans* configuration of the two variants in the patients could not be verified with certainty (due to the use of short read sequencing and the lack of a paternal sample), this segregation pattern is highly suggestive of an autosomal recessive inheritance in a compound heterozygous state. The variants were classified as pathogenic in these patients based on the ACMG criteria given in Supplementary 3. As predicted in Fig. 3, the mutant residue will inflict at least 4 clashing points with neighboring residues, which may affect the structure, and hence the function of the protein.

## Discussion

In a consanguineous Sudanese family, we identified two variants in *PLA2G6* in a compound heterozygous state in two patients with late onset parkinsonism. Pathogenic variants in this gene have been associated with autosomal recessive juvenile onset or early‐onset parkinsonism (OMIM #612953; PARK14). To our knowledge, this is the first case in which a *PLA2G6* variant is associated with late‐onset autosomal recessive presentation. Yet, it is still consistent with the complex nature of *PLA2G6*‐associated phenotypes and its tendency to feature multiple neurological disorders despite the rarity of its mutations.

The first variant we identified is a known pathogenic in‐frame deletion (NM_003560.4:c.2070_2072 del; p.Val691del) which, unlike our study, was reported in a homozygous state in all previous studies, and all reported individuals were cases of INAD.^24^, ^25^ *PLA2G6*‐associated INAD was also reported in another Sudanese family, where a homozygous pathogenic variant affecting a canonical splice site (NM_003560.2:c.1427+2T>C) was found in the two affected siblings.^26^

The second variant is also a known missense variant (NM_003560.4:c.956C>T; p.Thr319Met) located in exon 7, which was associated with several cases presenting with the same phenotype.^15^ A previous functional evaluation performed on fibroblasts from a patient carrying this variant—in a compound heterozygous state with p.His117Gln—showed a major palmitoylation defect.^27^

Apart from the unusually late age of onset, the presentation of the disease was consistent with the phenotypes of previous patients carrying p.Val691del or p.Thr319Met, and also with what is known about adult‐onset *PLA2G6*‐related parkinsonism in general. *PLA2G6*‐associated hippocampal changes have been previously described in number of previous studies.^28^ In this study, MRI of one proband but not the other showed hippocampal expansion. Hippocampal involvement was also described by Michelis and colleagues, reporting a novel *PLA2G6* mutation in a 35‐year‐old patient with adult‐onset autosomal recessive parkinsonism and early stages of dementia.^29^ In contrast, neither of our patients showed signs of dementia.

This study had limitations. Although one of our patients had a history of psychiatric manifestations, it was not feasible to perform a formal, detailed cognitive and psychiatric assessment. Also, iron deposition in the brain cannot be reliably ruled out as T2* and SWI MRI sequences were not obtained. Last, using a panel limits the chance of detecting any other possible molecular findings (e.g., intronic variants or coding variants in other genes not included in the panel) which are classically detectable with WES or WGS.

## Conclusion

We identified two *PLA2G6* variants in two patients from a consanguineous Sudanese family, consistent with a genetic diagnosis of autosomal recessive parkinsonism. Although both variants were previously reported, these patients had an unusually late onset of disease in the sixth decade of life, expanding the age range of the *PLA2G6*‐associated parkinsonism. Functional analysis of the effects of these variants on the structure and function of iPLA2β may unravel the mechanisms of this variability.

## Authors Contribution

Conception and design: UW, YB, CT, J‐C C, SL, OS, LEOE; Primary clinical evaluation: OS and YB. Secondary clinical assessment (UK Parkinson's Disease Society Brain Bank Clinical Diagnostic Criteria AND MDS‐UPDRS), participant recruitment and sampling: YB, MI, KE, and IE. DNA preparation and PCR: YB: Target NGS analysis: CT. Data analysis and interpretation: YB, CT, J‐C C, SL and UW. Writing the first draft: YB. Writing with critical revision: YB, UW, J‐C C, and LEOE. All authors revised and approved the final version of the manuscript.

## Conflict of Interest

The authors declare no conflict of interest.

## Funding Information

Program “Investissements d'Avenir” ANR‐10‐IAIHU‐06, and from a grant from France Parkinson: to J‐C C, SL, and CT. UKB: PSP‐Element A‐397.0003 mit der Kostenstelle 993000: for UW and YB. Princess Nourah bint Abdulrahman University Researchers supporting project number PNURSP2022R172, Princess Nourah bint Abdulrahman University, Riyadh, Saudi Arabia to LEOE.

## Supporting information


Appendix S1.

Appendix S2.

Appendix S3.

Figure S1.
