# Supplementary material for: PLA2G6 ‐associated late‐onset parkinsonism in a Sudanese family
Source: Ann Clin Transl Neurol. 2023 May 3;10(6):983–9. doi: 10.1002/acn3.51781 (PMC10270271; doi:10.1002/acn3.51781)
Supplement: Supplementary file 3 — Appendix S3. [file ACN3-10-983-s001.docx]

Variants classification according to American College of Medical Genetics and Genomics (ACMG)

| Variant & Classification | ACMG Evidence | Criteria | Description |
| --- | --- | --- | --- |
| *PLA2G6*:p.Val961del  Pathogenic | Strong | PS1 | Same amino acid change as a previously established pathogenic variant regardless of nucleotide change. |
|  | Strong | PS3 | Well-established in vitro or in vivo functional studies supportive of a damaging effect on the gene or gene product. |
|  | Moderate | PM2 | Low frequency in gnomAD. |
| PLA2G6:p.Thr361Met  Pathogenic | Supportive | PP1 | Cosegregation with disease in multiple affected family members in a gene definitively known to cause the disease |
| *PLA2G6*:p.Thr361Met  Likely Pathogenic | Moderate | PS1 | Same amino acid change as a previously established pathogenic variant regardless of nucleotide change. Downgraded to moderate because of conflicting VUS in ClinVar. |
|  | Moderate | PM2 | Low frequency in gnomAD. |
|  | Strong | PS3 | Well-established in vitro or in vivo functional studies supportive of a damaging effect on the gene or gene product. Downgraded to supporting because the evidence was obtained from studies of this variant in compound heterozygous state with another disease-causing variant. |
|  | Moderate | PM3 | For recessive disorders, detected in trans with a pathogenic variant. Downgraded to supporting because of the lack of paternal sample. |
|  | Supporting | PP1 | Cosegregation with disease in multiple affected family members in a gene definitively known to cause the disease |
|  | Supporting | PP3 | Multiple lines of computational evidence support a deleterious effect on the gene or gene product. |
|  | Very strong | PP5 | Reputable source recently reports variant as pathogenic, but the evidence is not available to the laboratory to perform an independent evaluation |

:
