# Supplementary figures and images for: PLA2G6 ‐associated late‐onset parkinsonism in a Sudanese family
Source: Ann Clin Transl Neurol. 2023 May 3;10(6):983–9. doi: 10.1002/acn3.51781 (PMC10270271; doi:10.1002/acn3.51781)

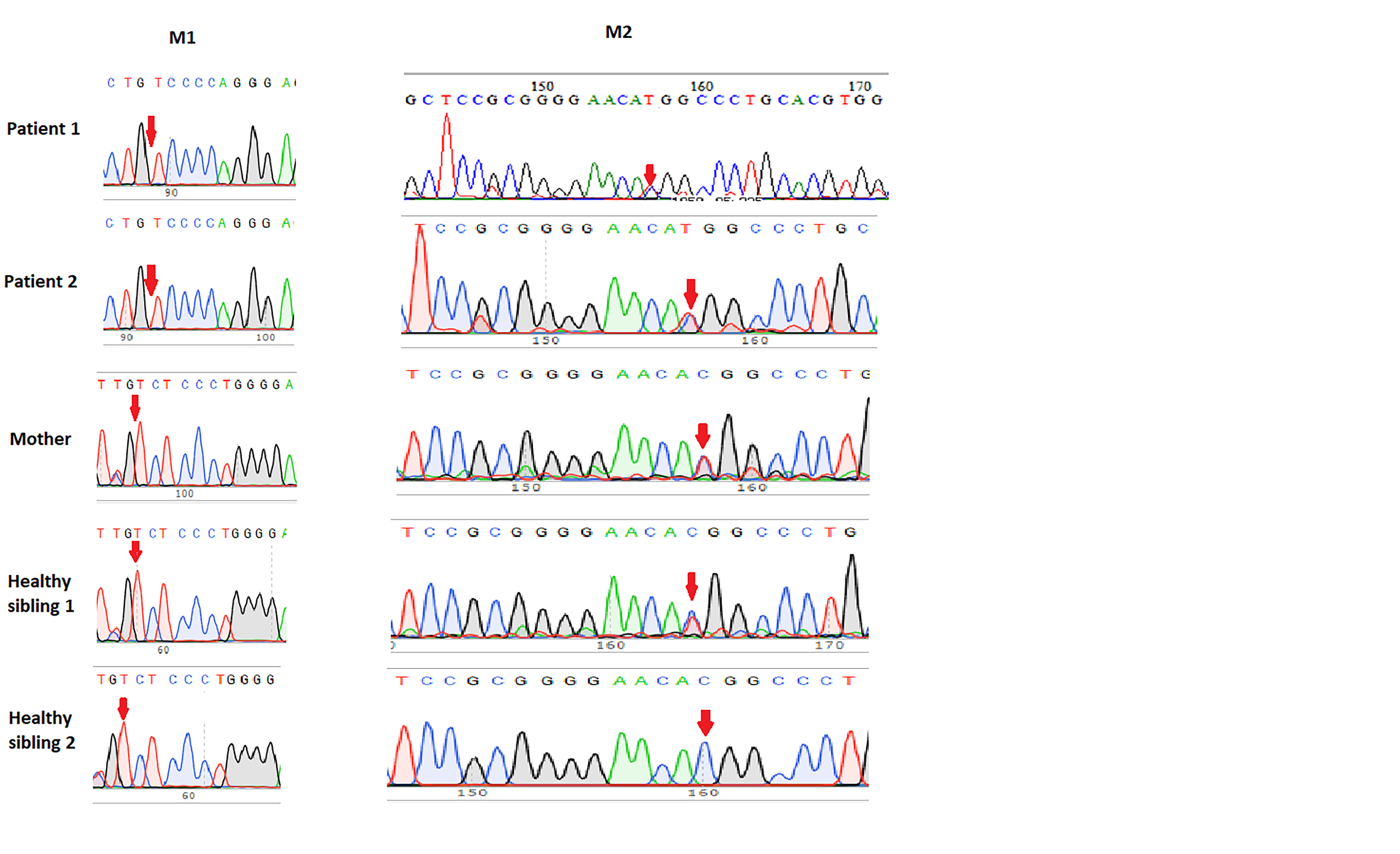

Supplement: Supplementary file 4 — Figure S1. [file ACN3-10-983-s004.tif]
